# Improved fed-batch bioprocesses using chemically modified amino acids in concentrated feeds

**DOI:** 10.1186/1753-6561-7-S6-P46

**Published:** 2013-12-04

**Authors:** Ronja Mueller, Isabell Joy-Hillesheim, Karima El Bagdadi, Maria Wehsling, Christian Jasper, Joerg von Hagen, Aline Zimmer

**Affiliations:** 1 Merck Millipore, Pharm-Chemical Solutions - Research & Development Upstream, Darmstadt, Germany; 2 Merck KGaA, Performance Materials - Advanced Technologies Synthesis, Darmstadt, Germany

## Background

Fed-batch culture bioprocesses are essential for the production of therapeutic proteins [1]. In these cultures, concentrated feeds are added during cultivation to prevent nutrient depletion and to extend the growth phase, thus increasing product concentration [2]. One limitation arises from the low solubility of some amino acids at high concentrations, in particular tyrosine [3]. This amino acid is commonly solubilized in separate feeds at basic pH [4] inducing pH spikes and precipitation when added in the bioreactor. This work describes the evaluation of several chemically modified tyrosines as alternative to simplify fed-batch bioprocesses by using single feeding strategies at neutral pH.

## Materials and methods

For solubility experiments, increasing concentrations of modified tyrosines were solubilized in Merck Millipore proprietary feed at pH 7,0 until reaching the maximum solubility. Stability was assessed during 6 months in Merck Millipore proprietary medium and amino acids (including modified tyrosine) were quantified by ultra performance liquid chromatography using ACQ·Tag Ultra reagent (Waters).

For batch cultures, modified tyrosines were solubilized at a concentration of 4,5 mM in Merck Millipore proprietary medium depleted in unmodified tyrosine. The control medium contained 1 mM tyrosine di-sodium salt. CHO-S cells were seeded at 1.10^5 ^cells/ml in 50 ml spin tubes and incubated at 37°C, 5% CO_2_, 80% humidity and a rotation speed of 320 rpm. Growth and viability were monitored during 11 days using Beckman Coulter ViCell^®^.

For fed-batch cultures, CHO-S cells expressing a human monoclonal antibody were seeded at 2.10^5 ^cells/ml in medium containing tyrosine di-sodium salt. Feeds were added every other day starting at day 3. In the control, tyrosine di-sodium salt was added in a separate feed at pH 11 whereas modified tyrosines were solubilized in the main feed at pH 7,0. Glucose was maintained at 4 g/L using a separate feed. Growth and viability were monitored during 14 days using Beckman Coulter ViCell^®^.

For antibody analysis, IgG concentrations were determined by a turbidometric method using Roche Cedex bio HT^®^. Intact mass analysis, peptide mapping and glycan analyses were performed on samples from day 14 using mass spectrometry and 2-aminobenzamide labeling followed by ultra performance liquid chromatography.

## Results

### Solubility and stability experiments

Chemically modified tyrosines demonstrated an increased solubility in concentrated feed at neutral pH in comparison with tyrosine or tyrosine di-sodium salt (Table 1). The highest solubility was achieved for the modified tyrosine 4 with a value of 75 g/L. The stability was assessed by quantification of the modified amino acid through ultra performance liquid chromatography. Moreover, no precipitation was detected over a 6 months period indicating that the chemical modification was stable in the tested conditions.

**Table 1:** Maximum solubility and stability of tyrosine derivates in Merck Millipore proprietary feed or medium at pH 7,0.

Molecule	Tyrosine	Tyrosine di-sodium salt	Modified Tyrosine 2	Modified Tyrosine 3	Modified Tyrosine 4
Solubility in concentrated feed at pH 7,0	Not soluble	< 1 g/l	10 g/l	70 g/l	75 g/l

Stability in medium at pH 7,0	-	> 6 months	> 6 months	> 6 months	> 6 months

### Batch and fed-batch cultures

The performance in batch culture was determined using tyrosine depleted media and supplementation with the different derivates. The growth of CHO-S cells with medium supplemented with modified tyrosine 2 reached only 50% of the growth of the control indicating that this molecule may not be able to be taken up by the cells or to promote growth through alternative mechanisms. This derivate was not evaluated further. Both modified tyrosines 3 and 4 induced a growth comparable to the control culture until day 6 and were then able to extend the growth during 2 additional days indicating that both derivates can be used successfully in batch cultures.

**Figure 1:**
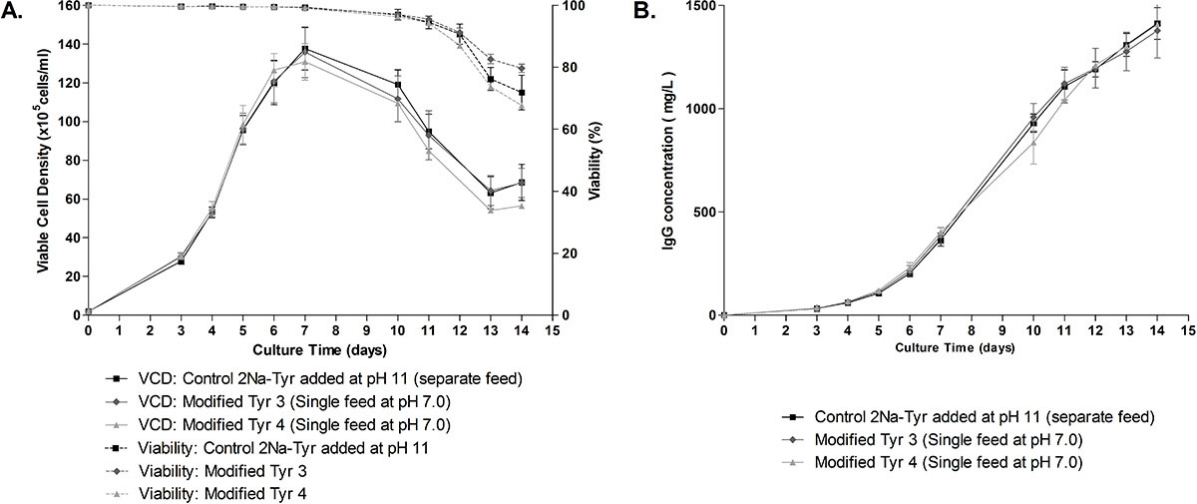
**Performance of the modified tyrosines in fed-batch culture**. A: Viable cell density and viability during the fed-batch process. B. IgG production during the fed-batch culture.

In fed-batch mode, modified tyrosines 3 and 4 were solubilized in a single concentrated feed at pH 7,0 and added to the culture every other day starting at day 3. The growth of recombinant CHO-S cells obtained with the derivates was similar to the control (where tyrosine di-sodium salt was added through a separate feed at pH 11) reaching a maximum viable cell density of 14.10^6 ^at day 7 (Figure 1A). The titer obtained after 14 days was equivalent in the two feeds and the new single feed process with final titers around 1,5 g/l (Figure 1B) indicating no negative effect of the chemical modification on productivity.

### Impact of modified tyrosines on the monoclonal antibody quality attributes

Intact mass, peptide mapping and glycosylation analyses were performed on the monoclonal antibody to study the impact of modified tyrosines on the final molecule. No significant difference could be established in either the intact mass of the antibody or the detailed analysis of the tryptic peptides by mass spectrometry. Glycosylation analysis indicated the same overall glycosylation pattern with 8,2% GlcNac3Man3Fuc, 72,3% G0F, 7,4% Man5 and 8,5% G1F glycans. Altogether these data indicated that the use of chemically modified tyrosines in concentrated feeds did not induce any detectable modification of the monoclonal antibody.

## Conclusions

The chemical modification of tyrosine can improve the solubility of the amino acid by up to 70 fold.

Modified tyrosines are stable in chemically defined media and feeds and can be used in batch and fed-batch mode. The use of these modified amino acids in fed-batch bioprocesses has no detectable impact on the monoclonal antibody or the recombinant protein produced. Altogether, this study demonstrates that modified amino acids can be used successfully in highly concentrated neutral feeds to improve and simplify next generation fed-batch processes.
